# Assessment of patient perception and administration technique of vaginal tablets at a tertiary care women's hospital

**DOI:** 10.1016/j.rcsop.2025.100632

**Published:** 2025-07-03

**Authors:** Nirmal Raj Marasine, Garima Kunwar, Manisha Chaudhary, Anjana Adhikari, Sabina Sankhi

**Affiliations:** a Department of Pharmacy, CiST College, Pokhara University, Kathmandu, Nepal; b Department of Pharmacy, Shree College of Technology, Bharatpur, Chitwan, Nepal

**Keywords:** Administration technique, Patient's perception, Reproductive health, Tertiary care women hospital, Vaginal tablets

## Abstract

**Background:**

Vaginal tablets offer an effective and patient-friendly route for both localized and systemic therapies, bypassing hepatic first-pass metabolism and minimizing gastrointestinal side effects. However, in low-resource settings like Nepal, their optimal use is often hindered by patient's perception and administration techniques.

**Objective:**

This study aimed to assess patient perception and administration technique of vaginal tablets at a tertiary women's hospital in Nepal.

**Methods:**

A hospital-based cross-sectional study was conducted from February to July 2024 among 117 women of reproductive age visiting a tertiary care women's hospital in Kathmandu. Data were collected through face-to-face interviews using a validated 8-item perception questionnaire and an 8-item administration technique checklist. Bivariate analysis using Pearson's chi-square test and binary logistic regression were performed to identify factors associated with patients' perceptions and administration techniques.

**Results:**

More than half (56.4 %) of the patient's demonstrated adequate administration techniques (scores ≥6), yet a striking 76.1 % exhibited suboptimal perceptions towards vaginal tablet use. Factors such as being unmarried, having lower educational attainment (illiterate or school level education), being unemployed or a housemaker, residing in rural areas, and lacking prior experience were significantly associated with poorer perceptions. Notably, younger age, prior use and counseling by pharmacists were positively linked to better administration techniques.

**Conclusion:**

Despite adequate administration practices among most women, suboptimal perceptions persist, influenced by demographic and experiential factors. Tailored, provider-led educational interventions focusing on counseling and user-friendly instructions are essential to enhance patient understanding, comfort, and adherence, thereby improving therapeutic outcomes and empowering women in their reproductive health decisions.

## 1 Background

The vaginal drug delivery system serves as an effective route for both localized and systemic therapies, with the advantage of the vaginal mucosa's dense vascularization and avoidance of hepatic first-pass metabolism and gastrointestinal interference.^1^ These formulations are widely used in the treatment of conditions such as vulvovaginal candidiasis (VVC), bacterial vaginosis (BV), trichomoniasis, and hormonal imbalances, including vaginal atrophy and progesterone supplementation in infertility treatments.^2^^,^^3^ VVC and BV are estimated to affect 75 % and 30 % of reproductive age group women at least once in their life, with trichomonas vaginalis (TV) affecting nearly 57–180 million people globally.^4^

Vaginal tablets are well known for their ease of use, capacity to deliver prolonged regimens via controlled-release mechanisms, and ability to ensure less frequent dosing and lower daily doses with reduced side effects.^3^ Additionally, the vaginal route offers a patient-friendly alternative to parenteral administration by avoiding pain, tissue injury, and infection risk, while allowing for convenient self-insertion and removal of the dosage form without clinical assistance.^1^^,^^2^ Despite their clinical utility, the effectiveness of vaginal tablets is significantly influenced by patients' perceptions of the treatment modality and adherence to proper administration techniques.^4^

The commonly prescribed vaginal tablet formulation in Nepal includes clotrimazole for fungal infection, metronidazole for bacterial vaginosis or trichomoniasis, and progesterone for hormonal support.^5^, ^6^, ^7^ In low- and middle-income countries, patient education on the proper use of vaginal dosage forms remains limited, often leading to subtherapeutic outcomes and an increased risk of local irritation, discomfort, or therapeutic failure.^5^ Particularly in South Asian contexts, cultural taboos, limited awareness of sexual and reproductive health, and deeply rooted sociocultural stigma further hinder the effective use of vaginal drug delivery systems.^6^

Improper administration techniques of vaginal tablets may lead to consequences such as inconsistent drug absorption, reduced efficacy, and an increased risk of adverse events, including vaginal discharge, irritation, and infection.^7^ These issues, in turn, influence patient adherence and satisfaction, which are two critical components of therapeutic success, particularly in chronic or recurrent conditions such as vulvovaginal candidiasis or bacterial vaginosis.^8^ Patients' prior experience with vaginal tablet use, interactions with healthcare providers, and underlying sociocultural beliefs shape their perceptions, significantly impacting therapeutic outcomes and contributing to both intentional and unintentional non-adherence.^9^

Despite the numerous benefits of vaginal drug delivery and vaginal tablets, this remains an underexplored topic in Nepal. There is an urgent need to investigate the extent to which women understand and correctly administer vaginal tablets, as well as how their perceptions influence treatment adherence. Existing global literature primarily focuses on the pharmacokinetic and pharmaceutical aspects of vaginal drug delivery, with limited attention to patient-centered outcomes or behavioral factors in low- and middle-income countries.^10^ To date, no published study has systematically assessed patient perceptions or administration techniques of vaginal tablets in Nepal, particularly within the setting of a tertiary-level gynecological hospital.

This study aims to fill a critical knowledge gap by evaluating both the perceptions and actual administration practices of women using vaginal tablets at a tertiary-level women's hospital in Nepal. The findings are expected to inform the development of educational and clinical interventions that enhance therapeutic outcomes and promote culturally sensitive gynecological care.

## 2 Methodology

### 2.1 Study design, setting and population

A hospital-based cross-sectional study was conducted over a six-month period from February to July 2024 at Paropakar Maternity and Women's Hospital, also known as Prasuti Griha located in Thapathali, Kathmandu. It is the first maternity hospital of Nepal. The hospital has 415 beds, of which 336 are allocated to inpatient care; 241 beds are dedicated for obstetrics, 61 for gynecology and 34 for neonatology. The hospital provides obstetrics and gynecology, neonatology, radiology, and pathology services.^11^ The study population included women of reproductive age (18–45 years) who visited the outpatient department and were either currently prescribed or had previously used vaginal tablets, and who were willing to provide informed consent. Patients who were unwilling to participate, had a known psychiatric illness, or were terminally ill and unable to be interviewed were excluded from the study.

### 2.2 Sample size and sampling techniques

The sample size for the study was calculated using the Cochrane formula: n = z^2^pq / e^2^, where n is the desired sample size, z is the standard normal variate (1.96 at 5 % types I error), p is the estimated prevalence, q is 1 – p, and e is the margin of error (5 %). According to the census of the gynecological outpatient department (OPD) at Paropakar Maternity and Women's Hospital, a total of 3000 gynecological outpatients visited the OPD during a one-month period (March 1 to March 30, 2024). Based on pharmacy records, 249 boxes of vaginal tablets were dispensed during that period, resulting in a prevalence (p) of 249/3000 = 0.083. Consequently, q = 1–0.083 = 0.917. Substituting these values into the formula: n = (1.96^2^ × 0.083 × 0.917) / (0.05^2^), the calculated sample size was 117.

### 2.3 Data collection

Face-to-face interviews were conducted in the Nepali language by undergraduate pharmacy students who received two days of comprehensive training on the study objectives and procedures. The questionnaire, developed based on previously published articles related to vaginal tablet perception and administration techniques,^1^^,^^12^, ^13^, ^14^, 15., 16. was initially prepared in English (attached as Supplementary File S1) and reviewed by two clinical pharmacists, one hospital pharmacist, and one gynecologist to ensure content validity. These experts assessed the relevance, clarity, and comprehensiveness of the items, and their feedback was used to refine the questionnaire. The finalized English version was then translated into Nepali, followed by back-translation by independent bilingual experts to ensure semantic equivalence. The Nepali version demonstrated good internal consistency (Cronbach's alpha = 0.83) in a pilot study involving 12 participants.

### 2.4 Outcome measures

The outcome of interest was patient perception and administration techniques. The 8-item five-point Likert scale was used to measure the patients perception towards vaginal tablets. This tool assesses various aspects of patients satisfaction with vaginal tablet use, including effectiveness of treatment, comfort with healthcare providers, convenience, acceptability of vaginal medication, preference of vaginal vs oral therapy, privacy during administration, proper education for administration, and confidence in effectiveness. Patients were asked to indicate their level of agreement with each of the eight items using a 5-point Likert scale ranging from 1 (strongly disagree) to 5 (strongly agree). The total score was calculated by summing the scores of the individual items and ranged from 8 to 40. The scores were dichotomized using a median-based cutoff: patients with scores <30 were categorized as having sub-optimal perception, while those with scores ≥30 were classified as having optimal perception. An 8-item checklist was used to assess patients' administration techniques of vaginal tablets. The checklist included key steps such as removing the applicator from the wrapper with clean hands, pulling out the plunger of the applicator, placing the thick end of the tablet into the applicator, lying down or taking a suitable position before insertion, inserting the applicator and releasing the tablet properly, discarding the tablet and using a new one if it fell, withdrawing the applicator gently and cleaning it, and safely storing the applicator for reuse. Each item was scored dichotomously as “Yes” (1 point) or “No” (0 points), yielding a cumulative score ranging from 0 to 8. Higher scores indicated greater compliance with recommended administration practices. Due to distribution of score, total scores were dichotomized into two categories: “inadequate administration technique” (<6) and “adequate administration technique” (≥6).

### 2.5 Explanatory variables

Explanatory variables considered in this study were age (categorized as 18–30, 31–40, and 41–49), marital status (ever married/married), religion (Hindu, Buddhism, Islam, and Christianity), ethnicity (Brahmin/Chhetri, Dalit, Janajati, Madhesi, and Muslim), educational status (illiterate, school level, intermediate, and bachelor or above), current occupation (unemployed, professional works, and homemakers), family type (nuclear/joint), family monthly income in Nepalese Rupees (NPR) (10,000–25,000; 26,000–50,000; 51,000–75,000; 76,000–1,00,000; and ≥ 1,00,000), residence (urban/rural), previous use of a vaginal tablet [first-time users (those who were prescribed vaginal tablets for the first time during the current outpatient visit and had no prior history of use — coded as “No”) and experienced users (those who had used vaginal tablets at least once prior to the current prescription — coded as “Yes”], reason for being prescribed a vaginal tablet (vaginal infection/hormonal therapy), and the person who explained how to use the vaginal tablet (doctor, nurse/health assistant, and pharmacist). Participants were asked whether they would be willing to use a vaginal tablet in the future (responses categorized as Yes or No), and reasons for willingness (better efficacy, fewer side effects, and comfortable application) and for not willingness (leakage insertion difficulties, discomfort/pain during use, sexual interference, need for privacy, less practical allergy/local irritation).

### 2.6 Statistical analysis

Descriptive statistics were computed to summarize the distribution of sociodemographic characteristics and outcome variables. Measures of central tendency (means and standard deviations) were used for continuous variables, while frequencies and percentages were reported for categorical variables. Bivariate analyses, including Chi-square tests and Fisher's exact tests (when expected cell counts were less than five), were performed to examine associations between explanatory variables and the outcome variables. Variables found to be statistically significant in the bivariate analysis were included in the final logistic regression model. The model included all relevant variables, and both unadjusted and adjusted odds ratios (ORs), along with their 95 % confidence intervals (CIs), are reported. The variance inflation factor (VIF) was calculated for the selected independent variables to assess multicollinearity, and VIF were between 1 and 2. A VIF greater than five was considered indicative of multicollinearity. Statistical significance was determined using a two-sided *p*-value threshold of <0.05. All data analyses were conducted using R (version 4.0.4).

### 2.7 Ethical considerations

The study was conducted in accordance with the Declaration of Helsinki and was approved by the Institutional Review Committee of Paropakar Maternity and Women's Hospital (Ref. No: 64/1557). Participation was entirely voluntary, and written informed consent was obtained from each patient prior to data collection. For illiterate patients, thumbprint impressions were obtained in lieu of signatures, with prior approval from the ethics committee. All patients were informed about the study's objectives, and they were assured that they could access their data after collection.

## 3 Results

The baseline characteristics and bivariate analyses by perception and administration technique of vaginal tablets are presented in Table 1. Among the total participants (*n* = 117), nearly half were aged 18–30 years (47.9 %), the majority were ever married (82.9 %) and identified as Hindu (82.9 %). More than half of the participants belonged to the Brahmin/Chhetri caste (52.1 %), followed by Janajati (34.2 %) and Dalit (13.7 %). Regarding education, 35.9 % had completed school-level education, 29.9 % had intermediate-level qualification, and 17.1 % were illiterate. More than half of the participants were homemakers (55.6 %), while 26.5 % were professionals and 17.9 % were unemployed. Most participants resided in urban areas (77.8 %) and came from nuclear families (59.8 %). Regarding vaginal tablet use, 61.5 % of participants reported previous use, primarily for treating vaginal infections (70.9 %), followed by hormonal therapy (29.1 %). The majority received counseling on administration from pharmacists (63.2 %). More than half (56.4 %) of the patients demonstrated adequate administration techniques, 76.1 % held suboptimal perceptions regarding vaginal tablet use.

**Table 1:** Baseline characteristics and bivariate analyses by perception and administration techniques.

Characteristics	Total	Perception			Administration technique		
	(*n* = 117;100 %)	Optimal (*n* = 28;23.9 %)		Sub-optimal (*n* = 89;76.1 %)	Adequate (*n* = 66;56.4 %)	Inadequate (*n* = 51;43.5)	
	n (%)	n (%)	n (%)	p-value	n (%)	n (%)	p-value
**Age (Yrs)**							
18–30	56 (47.9)	10 (35.7)	46(51.6)	0.045	30 (45.5)	26 (51.0)	0.040
31–40	33 (28.2)	8 (28.6)	25(28.1)		20 (30.3)	13 (25.5)	
41–45	28 (23.9)	10 (35.7)	18(20.3)		16 (24.2)	12 (23.5)	
Mean ± S.D. 32.62 ± 8.184							
**Marital Status**							
Ever Married	97 (82.9)	19 (67.8)	78 (87.6)	0.021^a^	56 (84.8)	41 (80.4)	0.421^a^
Never Married	20 (17.1)	9 (32.2)	11 (12.4)		10 (15.2)	10 (19.6)	
**Religion**							
Hindu	97 (82.9)	23 (82.2)	74 (83.1)	0.511	55 (83.3)	42 (82.4)	0.292
Buddhism	6 (5.1)	2 (7.1)	4 (4.5)		4 (6.1)	2 (3.9)	
Islam	4 (3.4)	1 (3.6)	3 (3.4)		3 (4.5)	1 (2.0)	
Christianity	10 (8.5)	2 (7.1)	8 (9.0)		4 (6.1)	6 (11.7)	
**Ethnicity**							
Brahmin/Chhetri	61 (52.1)	13 (46.4)	48 (54.0)	0.370	36 (54.5)	25 (49.1)	0.521
Dalit	6 (5.1)	1 (3.6)	5 (5.6)		3 (4.5)	3 (5.9)	
Janajati*	40 (34.1)	10 (35.7)	30 (33.7)		22 (33.4)	18 (35.2)	
Madhesi	7 (6.0)	3 (10.7)	4 (4.5)		3 (4.5)	4 (7.8)	
Muslim	3 (2.6)	1 (3.6)	2 (2.2)		2 (3.1)	1 (2.0)	
**Educational Qualification**							
Illiterate	20 (17.1)	2 (7.2)	18 (20.2)	0.003	8 (12.2)	12 (23.5)	0.102
School level	42 (35.9)	6 (21.4)	36 (40.5)		20 (30.3)	22 (43.1)	
Intermediate	35 (29.9)	10 (35.7)	25 (28.1)		21 (31.8)	14 (27.5)	
Bachelor or above	20 (17.1)	10 (35.7)	10 (11.2)		17 (25.7)	3 (5.9)	
**Current occupation**							
Unemployed	21 (17.9)	2 (7.2)	19 (21.3)	0.049	9 (13.7)	12 (23.5)	0.215
Professional Works	31 (26.5)	16 (57.1)	15(16.9)		21 (31.8)	10 (19.6)	
Homemakers	65 (55.6)	10 (35.7)	55 (61.8)		36 (54.5)	29 (56.9)	
**Family Type**							
Nuclear	70 (59.8)	16 (57.2)	54 (60.7)	0.032^a^	38 (57.6)	32 (62.7)	0.361^a^
Joint	47 (40.2)	12 (42.8)	35 (39.3)		28 (42.4)	19 (37.3)	
**Family Income (NRs)**							
10,000-25,000	17 (14.5)	3 (10.7)	14 (15.7)	0.204	8 (12.1)	9 (17.7)	0.349
26,000-50,000	64 (54.7)	13 (46.4)	51 (57.3)		34 (51.5)	30 (58.8)	
51,000-75,000	13 (11.1)	4 (14.3)	9 (10.1)		9 (13.6)	4 (7.8)	
76,000-100,000	17 (14.5)	5 (17.9)	12 (13.5)		10 (15.2)	7 (13.7)	
≥ 100,000	6 (5.1)	3 (10.7)	3 (3.4)		5 (7.6)	1 (2.0)	
**Residence**							
Urban	91 (77.8)	17 (60.7)	74 (83.2)	0.015^a^	54 (81.8)	37 (72.5)	0.149^a^
Rural	26 (22.2)	11 (39.3)	15 (16.8)		12 (18.2)	14 (27.5)	
**Have you ever used a vaginal tablet before**							
Yes	72 (61.5)	10 (35.7)	62 (69.7)	<0.001^a^	28 (42.4)	44 (86.3)	<0.001^a^
No	45 (38.5)	18 (64.3)	27 (30.3)		38 (57.6)	7 (13.7)	
**Why were you prescribed a vaginal tablet?**							
Vaginal Infection	83 (70.9)	15 (53.6)	68 (76.4)	0.026^a^	40 (60.6)	43 (84.3)	0.003^a^
Hormonal Therapy	34 (29.1)	13 (46.4)	21 (23.6)		26 (39.4)	8 (15.7)	
**Counseling by**							
Doctor	43(36.7)	6 (21.4)	37(41.6)	<0.001	17(25.7)	26 (51.0)	<0.001
Pharmacist	74 (63.2)	22(78.6)	52(58.4)		49(74.3)	25(49.0)	

Notes: Janajati: Newar, Magar, Gurung, Tamang, Sherpa; Professional work: students, private/government job, business; Ever married: married, divorced, separated, widow, a-value for chi-square test; rest of the p values from Fisher's Exact test.

Bivariate analyses revealed several significant associations between various factors and the perception and administration technique of vaginal tablets. Patients perceptions significantly vary by age, marital status, education, occupation, family type, and place of residence. Previous use of vaginal tablets was strongly associated with both perception and administration technique (*p* < 0.001). Participants using vaginal tablets for hormonal therapy had significantly poorer perception and inadequate administration technique (*p* = 0.026 and *p* = 0.003, respectively). Receiving instructions from pharmacists was also linked to sub-optimal perception and adequate technique (*p* < 0.001). No statistically significant relationship was observed with religion, ethnicity, or monthly family income.

Table 2 presents majority of patients believed vaginal tablets were effective in treating their condition (70.1 %) and felt comfortable discussing them with healthcare providers (68.4 %). Most of the patients also found vaginal tablets convenient to use (69.2 %) and acceptable in terms of vaginal insertion (79.5 %). Regarding preference, 55.5 % favored vaginal tablets over oral therapy, though 18.8 % disagreed. Privacy during administration was perceived positively by 68.3 % of patients, while 7.7 % disagreed. Additionally, most patients felt well-educated about the purpose and administration of vaginal tablets (69.3 %) and expressed confidence in their effectiveness (76.9 %).

**Table 2:** Patients perception towards vaginal tablets (n = 117).

No	Statement	SDa	Da	N	A	Sa
P1	Vaginal tablets are effective in treating my condition.	5 (4.3 %)	10 (8.5 %)	20 (17.1 %)	40 (34.2 %)	42 (35.9 %)
P2	I feel comfortable discussing vaginal tablets with healthcare providers.	7 (6.0 %)	12(10.3 %)	18 (15.4 %)	45(38.5 %)	35 (29.9 %)
P3	Vaginal tablets are convenient to use.	3 (2.6 %)	8 (6.8 %)	25 (21.4 %)	55 (47.0 %)	26 (22.2 %)
P4	The idea of inserting medication vaginally is acceptable to me.	4 (3.4 %)	5 (4.3 %)	15 (12.8 %)	54 (46.2 %)	39 (33.3 %)
P5	I prefer vaginal tablets over oral therapy for vaginal conditions.	10 (8.5 %)	12 (10.3 %)	30 (25.6 %)	35 (29.9 %)	30 (25.6 %)
P6	I have enough privacy during administration.	5 (4.3 %)	8 (6.8 %)	24 (20.5 %)	50 (42.7 %)	30 (25.6 %)
P7	I am properly educated about the purpose and administration of vaginal tablets.	3 (2.6 %)	6 (5.1 %)	27 (23.1 %)	43 (36.8 %)	38 (32.5 %)
P8	I feel confident that vaginal tablets work effectively.	4 (3.4 %)	8 (6.8 %)	15 (12.8 %)	49 (41.9 %)	41 (35.0 %)

SDa: Strongly disagree; Da: Disagree; N: Neutral; A: Agree; Sa: Strongly agree.

Among the participants, 72.6 % removed the applicator from the wrapper with clean hands, and 76.9 % pulled out the plunger of the applicator. However, only 55.6 % of patients correctly placed the thick end of the tablet into the applicator. Regarding positioning, 85.5 % of patients ensured they were lying down or in a suitable position before insertion, while 64.1 % correctly inserted the applicator and released the tablet. When the tablet fell, 51.3 % of patients discarded it and used a new one, whereas 48.7 % did not. Additionally, 68.4 % of patients withdrew the applicator gently and cleaned it afterward, and 59.8 % stored the applicator safely for reuse. The mean ± SD score was 5.64 ± 0.49, as shown in Table 3.

**Table 3:** Patient responses on vaginal tablet administration technique (*n* = 117).

S. N	Administration steps	Yes n (%)	No n (%)
1.	Did you remove the applicator from the wrapper with clean hands?	85 (72.6)	32 (27.4)
2.	Did you pull out the plunger of the applicator?	90(76.9)	27 (23.1)
3.	Did you place the thick end of the tablet into the applicator?	65 (55.6)	52 (44.4)
4.	Did you lie down or take a suitable position before insertion?	100 (85.5)	17 (14.5)
5.	Did you insert the applicator and release the tablet properly?	75 (64.1)	42 (35.9)
6.	If the tablet fell, did you discard it and use a new one?	60 (51.3)	57 (48.7)
7.	Did you withdraw the applicator gently and clean it?	80 (68.4)	37 (31.6)
8.	Did you store the applicator safely for reuse?	70 (59.8)	47(40.2)
	**Mean ± SD**	5.64 ± 0.49	

Table 4 shows the results of both unadjusted and adjusted logistic regression analyses examining the association between baseline characteristics and patients' perceptions towards vaginal tablets. Significant associations were observed with marital status, educational qualification, occupation, residence, reason for use, and prior experience with vaginal tablets. Individuals who had never been married had over three times (adjusted OR: 3.28; 95 % CI: 1.13–8.97) the odds of having a suboptimal perception compared to those who had ever been married. Likewise, women who were illiterate (adjusted OR: 0.09; 95 % CI: 0.04–0.98) or had only completed school level education (adjusted OR: 0.16; 95 % CI: 0.05–0.61) were more likely to report suboptimal perception. Unemployed individuals (adjusted OR:0.7; 95 % CI: 0.05–0.69) and homemakers (adjusted OR: 0.14; 95 % CI: 0.09–0.48) were more likely to exhibit poor perceptions compared to their employed counterparts. Additionally, those residing in rural areas (adjusted OR: 3.11; 95 % CI: 1.12–7.88) had significantly higher odds of suboptimal perception than urban residents. Furthermore, participants with no prior experience using vaginal tablets (adjusted OR: 3.89; 95 % CI: 1.24–9.71) had significantly higher odds of suboptimal perception compared to those with prior experience.

**Table 4:** Unadjusted and adjusted odds ratios (ORs) for factors associated with perception towards vaginal tablets.

Associated factors	Unadjusted OR [95 % CI]	p-value	Adjusted OR [95 % CI]	p-value
**Age (Years)**				
41–45	Ref.		Ref.	
31–40	0.58 [0.19–1.76]	0.431	0.53 [0.22–2.11]	0.451
18–30	0.39 [0.14–1.10]	0.075	0.31 [0.16–1.17]	0.080
**Marital Status**				
Ever Married	Ref.		Ref.	
Never Married	3.36 [1.24–9.11]	0.018*	3.28 [1.13–8.97]	<0.001**
**Educational Qualification**				
Bachelor or above	Ref.		Ref.	
Intermediate	0.40[0.13–1.25]	0.126	0.38 [0.31–2.32]	0.218
School level	0.17 [0.04–0.65]	0.010*	0.16 [0.05–0.61]	0.022*
Illiterate	0.11[0.02–0.89]	0.036*	0.09 [0.04–0.98]	0.030*
**Occupation**				
Professional work	Ref.		Ref.	
Homemakers	0.17 [0.05–0.52]	0.003*	0.14 [0.09–0.48]	0.002*
Unemployed	0.10 [0.02–0.72]	0.026*	0.7 [0.05–0.69]	0.018*
**Family Type**				
Nuclear	Ref.		Ref.	
Joint	1.16 [0.52–2.56]	0.719	1.19 [0.45–2.39]	0.541
**Residence**				
Urban	Ref.		Ref.	
Rural	3.19 [1.25–8.17]	0.015*	3.11 [1.12–7.88]	<0.001**
**Reason for Use**				
Vaginal Infection	Ref.		Ref.	
Hormonal Therapy	2.81 [0.15–6.85]	0.518	2.74 [0.97–5.76]	0.672
**Counseling by**				
Pharmacist	Ref.		Ref.	
Doctor	0.38 [0.14–1.04]	0.060	0.32 [0.19–1.91]	0.198
**Ever Used Vaginal Tablet**				
Yes	Ref.		Ref.	
No	4.14 [1.69–10.12]	0.002*	3.89 [1.24–9.71] *	<0.001**

OR = Odds Ratio; CI = Confidence Interval; * = p-value < 0.05, ** = p-value < 0.01, Ref = Reference group.

Table 5 shows the unadjusted and adjusted logistic regression analyses identifying factors associated with the administration technique of vaginal tablets. Women aged 41–45 years were more likely to demonstrate inadequate administration (aOR:1.11; 95 % CI: 1.01–1.45) compared to younger ones. Individuals with no prior experience using vaginal tablets had significantly lower odds (adjusted OR:0.10, 95 % CI: 0.03–0.27) of demonstrating adequate administration technique. Furthermore, counseling provided by doctors (adjusted OR:2.90, 95 % CI:1.22–5.72) was significantly associated with higher odds of inadequate administration technique when compared to counseling provided by pharmacists.

**Table 5:** Unadjusted and adjusted odds ratios (ORs) for factors associated with administration technique.

Associated Factors	Unadjusted OR (95 % CI)	p-value	Adjusted OR (95 % CI)	p-value
**Age (Years)**				
18–30	Ref.		Ref.	
31–40	1.33 [0.56–3.19]	0.519	1.15 [0.49–2.82]	0.426
41–45	1.16 [1.04–2.88]	0.012*	1.11 [1.01–1.45]	0.021*
**Ever Used Vaginal Tablet**				
Yes	Ref.		Ref.	
No	0.12[0.05–0.30]	<0.001**	0.10[0.03–0.27]	0.001**
**Why Prescribed Vaginal Tablet?**				
Vaginal Infection	Ref.		Ref.	
Hormonal Therapy	0.29 [0.12–1.70]	0.341	0.25[0.11–1.68]	0.439
**Counseling by**				
Pharmacist	Ref.		Ref.	
Doctor	3.00 [1.38–6.51]	0.005**	2.90 [1.22–5.72]	<0.001**

OR = Odds Ratio; CI = Confidence Interval; * = p-value < 0.05, ** = p-value < 0.01, Ref = Reference group.

## 4 Discussion

Optimal patient perception and correct administration of vaginal tablets are critical for ensuring effective therapeutic outcomes, particularly in managing conditions such as vulvovaginal candidiasis, bacterial vaginosis, and hormonal imbalances. This study provides valuable insights into women's perceptions and administration practices related to vaginal tablet use at a tertiary-level women's hospital in Nepal. Findings revealed that, while more than half of patients demonstrated adequate administration techniques, overall perceptions remained suboptimal.

Although over two-thirds of patients acknowledged the clinical effectiveness and convenience of vaginal tablets, nearly three-fourths exhibited suboptimal perception. This was especially evident among unmarried individuals, those from rural areas, and women without prior experience using vaginal tablets. Previous studies suggest that sociocultural taboos, lower health literacy, limited access to counseling, and discomfort in discussing genital health factors contribute to negative perceptions.^5^^,^^17^^,^^18^

Health beliefs and self-efficacy play a pivotal role in shaping medication attitudes and adherence in women's reproductive health. In this study, women with lower educational backgrounds, illiteracy, unemployment, and homemaker status were more likely to exhibit poor perception, highlighting the influence of socioeconomic vulnerability, limited autonomy, and lower health empowerment on the understanding and acceptance of vaginal therapies.

Although the mean administration technique score (5.64 ± 0.49) was found acceptable, notable gaps remained in several critical steps. Only 55.6 % correctly placed the tablet into the applicator, and improper practices were observed in hygienic disposal and reuse. Alarmingly, nearly half of the patients did not discard a dropped tablet, increasing the risk of infection and reduce therapeutic efficacy. These findings align with global literature, which emphasize that improper insertion, poor hygiene, and limited understanding of dosage regimens can significantly undermine the effectiveness of vaginal therapies.^19^^,^^20^

Prior use of vaginal tablets emerged as a strong predictor of both improved perception and correct administration technique, underscoring the importance of experiential learning in building confidence and competence in self-administered therapies.^21^ Notably, younger individuals (18–30 years) exhibited adequate administration techniques, likely due to their receptiveness to pharmacist counseling, enhanced cognitive and motor skills, and adaptability in managing their reproductive health. Their increased exposure to health information and willingness to learn further contributed to correct techniques, even when perception remained suboptimal. The association between pharmacist-led counseling and better administration practices reinforces the critical role of pharmacists in delivering accessible, informed guidance on vaginal product use.

While most patients expressed a willingness to use vaginal tablets in the future, they reported several barriers such as insertion difficulties, pain or discomfort, the need for privacy, and perceived impracticality (see Supplementary File S2). These concerns reflect deeper systemic challenges, including lack of confidential counseling environments, insufficient patient-provider communication, and deeply ingrained cultural norms regarding female reproductive health in South Asia. A systematic review supports that patient acceptability of vaginal products is influenced not only by clinical efficacy but also by socio-cultural factors and their ease of use, which are crucial for adherence and treatment success.^22^ Similarly, a study from Portugal revealed leakage (84.8 %), insertion difficulty (58.4 %), and discomfort (40.7 %) as the most common drawbacks of vaginal products.^1^ Concerns such as local irritation and sexual interference further emphasize the need for individualized counseling and user-centered product design.

Patient perceived impracticality related to vaginal tablets likely stems from the complexity of administration, time consumption, need for specific positioning, and disruption of daily routines. These issues can be particularly burdensome in settings with limited privacy or hygiene facilities, highlighting the importance of considering patients lifestyles and contextual realities when recommending vaginal formulations.

Interestingly, variables such as ethnicity, religion, family type, and income-related differences did not show significant associations with perception or technique. This may reflect either a relatively homogeneous patient population in terms of healthcare access or signal that barriers are more closely tied to educational, informational and experiential factors than to broader socioeconomic or cultural factors.

This study is among the first in Nepal to comprehensively evaluate both patient perceptions and administration techniques related to vaginal tablet use, a clinically significant yet underexplored aspect of women's health. The use of rigorous methodology and culturally validated data collection tools enhances the credibility of the findings. However, several limitations must be acknowledged. As a single-center study conducted in a tertiary care setting, the findings may not be fully generalizable to rural populations or primary healthcare contexts. Women in such settings often face greater barriers, such as lower health literacy, inadequate access to skilled healthcare professionals, and limited exposure to structured counseling, especially from pharmacists. Sociocultural stigma and lack of privacy may further discourage proper use and acceptance of vaginal therapies, contributing to lower adherence and suboptimal treatment outcomes. Additionally, the cross-sectional design limits causal interpretation, and reliance on self-reported data introduces potential recall and social desirability biases. The exclusion of terminally ill or psychiatric patients may have led to selection bias, possibly underrepresenting more vulnerable subgroups.

## 5 Conclusion

This study highlights a critical gap between women's adequate administration techniques and their suboptimal perceptions of vaginal tablet use. Prior experience with vaginal tablets positively influenced both perceptions and administration technique. Perceptions were further impacted by sociodemographic factors such as marital status, educational level, occupation, and rural residence, while younger age and pharmacist counseling emerged as a key drivers of improved administration practices. These findings emphasize the urgent need for tailored, provider-led educational interventions that foster patient understanding, confidence, and adherence. Strengthening communication between patients and healthcare professionals, along with clear, accessible instructions, can significantly enhance therapeutic outcomes and empower women in managing their reproductive health.

## CRediT authorship contribution statement

**Nirmal Raj Marasine:** Writing – review & editing, Writing – original draft, Visualization, Validation, Resources, Project administration, Methodology, Investigation, Formal analysis, Data curation, Conceptualization. **Garima Kunwar:** Writing – review & editing, Resources, Project administration, Data curation. **Manisha Chaudhary:** Writing – review & editing, Resources, Project administration, Data curation. **Anjana Adhikari:** Writing – review & editing, Resources, Project administration, Data curation. **Sabina Sankhi:** Writing – review & editing, Writing – original draft, Validation, Resources, Project administration, Methodology, Formal analysis.

## Declaration of competing interest

The author(s) declared no potential conflicts of interest with respect to the research, authorship, and/or publication of this article.

## Data Availability

The raw data used to support the findings of this study are made available from the corresponding author upon reasonable request.

## Appendix A Supplementary data

Supplementary material 1 Image 1

Supplementary material 2 Image 2
