# Supplementary material for: Assessment of patient perception and administration technique of vaginal tablets at a tertiary care women's hospital
Source: Explor Res Clin Soc Pharm. 2025 Jul 3;19:100632. doi: 10.1016/j.rcsop.2025.100632 (PMC12257023; doi:10.1016/j.rcsop.2025.100632)
Supplement: Supplementary file 1 — Supplementary material 1 [file mmc1.docx]

**Supplementary file 1**

**Data Collection Form**

**Assessment of Patient Perception and Administration Technique of Vaginal Tablets**

| Variable | Code | Response Options | Notes |
| --- | --- | --- | --- |
| 1. Participant ID | PID | Unique ID | e.g., 001, 002… |
| 2. Age (in years) | AGE | ___ years | Write actual age |
| 3. Age Group | AGEGP | 1 = 18–30 2 = 31–40 3 = ≥41 |  |
| 4. Marital Status | MARSTAT | 1 = Ever Married 2 = Never Married | Ever married includes divorced, separated, widow |
| 5. Religion | RELIGION | 1 = Hindu 2 = Buddhism 3 = Islam 4 = Christianity |  |
| 6. Ethnicity | ETHNICITY | 1 = Brahmin/Chhetri 2 = Dalit 3 = Janajati 4 = Madhesi 5 = Muslim | Janajati includes Newar, Magar, Tamang, etc. |
| 7. Educational Qualification | EDU | 1 = Illiterate 2 = School level 3 = Intermediate 4 = Bachelor or above |  |
| 8. Occupation | OCCUP | 1 = Unemployed 2 = Homemaker 3 = Professional (student, business, job) |  |
| 9. Family Type | FAMTYPE | 1 = Nuclear 2 = Joint |  |
| 10. Monthly Family Income (NPR) | INCOME | 1 = 10k–25k 2 = 26k–50k 3 = 51k–75k 4 = 76k–100k 5 = ≥100k |  |
| 11. Residence | RESIDENCE | 1 = Urban 2 = Rural |  |

**Have you ever used a vaginal tablet before?**
☐ Yes
☐ No

**Why were you prescribed a vaginal tablet?**
☐ Vaginal Infection
☐ Hormonal Therapy

**Who provided counseling/instructions on vaginal tablet use?**
☐ Doctor
☐ Pharmacist

**Patients perception towards vaginal tablets**

| **No.** | **Statement** | **SDa** | **Da** | **N** | **A** | **Sa** |
| --- | --- | --- | --- | --- | --- | --- |
| **P1** | Vaginal tablets are effective in treating my condition. |  |  |  |  |  |
| **P2** | I feel comfortable discussing vaginal tablets with healthcare providers. |  |  |  |  |  |
| **P3** | Vaginal tablets are convenient to use. |  |  |  |  |  |
| **P4** | The idea of inserting medication vaginally is acceptable to me. |  |  |  |  |  |
| **P5** | I prefer vaginal tablets over oral therapy for vaginal conditions. |  |  |  |  |  |
| **P6** | I have enough privacy during administration. |  |  |  |  |  |
| **P7** | I am properly educated about the purpose and administration of vaginal tablets. |  |  |  |  |  |
| **P8** | I feel confident that vaginal tablets work effectively. |  |  |  |  |  |

*SDa: Strongly disagree; Da: Disagree; N: Neutral; A: Agree; Sa: Strongly agree*

**Patient responses on vaginal tablet administration technique**

| **S. N** | **Administration steps** | **Yes** | **No** |
| --- | --- | --- | --- |
| 1. | Do you remove the applicator from the wrapper with clean hands? |  |  |
| 2. | Do you pull out the plunger of the applicator? |  |  |
| 3. | Do you place the thick end of the tablet into the applicator? |  |  |
| 4. | Do you lie down or take a suitable position before insertion? |  |  |
| 5. | Do you insert the applicator and release the tablet properly? |  |  |
| 6. | If the tablet fell, did you discard it and use a new one? |  |  |
| 7. | Do you withdraw the applicator gently and clean it? |  |  |
| 8. | Do you store the applicator safely for reuse? |  |  |

**Patient Preferences towards vaginal tablet**

| **Characteristics** | **Category** | **Option** |
| --- | --- | --- |
| Willing to use a vaginal tablet in future | Yes |  |
|  | No |  |
| Reasons for willingness to use vaginal tablet* | Better efficacy |  |
|  | Fewer side effects |  |
|  | Comfortable application |  |
| Reason for unwillingness to use vaginal tablet* | Leakage |  |
|  | Insertion difficulties |  |
|  | Discomfort/Pain during use |  |
|  | Sexual Interference |  |
|  | Need for privacy |  |
|  | Less practical |  |
|  | Allergy/local irritation |  |
