# Supplementary material for: Assessment of patient perception and administration technique of vaginal tablets at a tertiary care women's hospital
Source: Explor Res Clin Soc Pharm. 2025 Jul 3;19:100632. doi: 10.1016/j.rcsop.2025.100632 (PMC12257023; doi:10.1016/j.rcsop.2025.100632)
Supplement: Supplementary file 2 — Supplementary material 2 [file mmc2.docx]

**Table : Patient Preferences towards vaginal tablet (n=117)**

| **Characteristics** | **Category** | **n (%)** |
| --- | --- | --- |
| Willing to use a vaginal tablet in future | Yes | 76(65.0) |
|  | No | 41(35.0) |
| Reasons for willingness to use vaginal tablet* | Better efficacy | 11(14.47) |
|  | Fewer side effects | 13(17.10) |
|  | Comfortable application | 67(88.15) |
| Reason for unwillingness to use vaginal tablet* | Leakage | 15(36.58) |
|  | Insertion difficulties | 41(100.0) |
|  | Discomfort/Pain during use | 37(90.24) |
|  | Sexual Interference | 7(17.07) |
|  | Need for privacy | 41(100.0) |
|  | Less practical | 22(53.65) |
|  | Allergy/local irritation | 14(34.14) |
